# Whole-Body Analysis of a Viral Infection: Vascular Endothelium is a Primary Target of Infectious Hematopoietic Necrosis Virus in Zebrafish Larvae

**DOI:** 10.1371/journal.ppat.1001269

**Published:** 2011-02-03

**Authors:** Marion Ludwig, Nuno Palha, Corinne Torhy, Valérie Briolat, Emma Colucci-Guyon, Michel Brémont, Philippe Herbomel, Pierre Boudinot, Jean-Pierre Levraud

**Affiliations:** 1 Macrophages et Développement de l'Immunité, Institut Pasteur, Paris, France; 2 CNRS URA2578, Paris, France; 3 Virologie et Immunologie Moléculaire, INRA, Jouy-en-Josas, France; University of Washington, United States of America

## Abstract

The progression of viral infections is notoriously difficult to follow in whole organisms. The small, transparent zebrafish larva constitutes a valuable system to study how pathogens spread. We describe here the course of infection of zebrafish early larvae with a heat-adapted variant of the Infectious Hematopoietic Necrosis Virus (IHNV), a rhabdovirus that represents an important threat to the salmonid culture industry. When incubated at 24°C, a permissive temperature for virus replication, larvae infected by intravenous injection died within three to four days. Macroscopic signs of infection followed a highly predictable course, with a slowdown then arrest of blood flow despite continuing heartbeat, followed by a loss of reactivity to touch and ultimately by death. Using whole-mount *in situ* hybridization, patterns of infection were imaged in whole larvae. The first infected cells were detectable as early as 6 hours post infection, and a steady increase in infected cell number and staining intensity occurred with time. Venous endothelium appeared as a primary target of infection, as could be confirmed in *fli1:GFP* transgenic larvae by live imaging and immunohistochemistry. Disruption of the first vessels took place before arrest of blood circulation, and hemorrhages could be observed in various places. Our data suggest that infection spread from the damaged vessels to underlying tissue. By shifting infected fish to a temperature of 28°C that is non-permissive for viral propagation, it was possible to establish when virus-generated damage became irreversible. This stage was reached many hours before any detectable induction of the host response. Zebrafish larvae infected with IHNV constitute a vertebrate model of an hemorrhagic viral disease. This tractable system will allow the *in vivo* dissection of host-virus interactions at the whole organism scale, a feature unrivalled by other vertebrate models.

## Author Summary

The zebrafish larva is uniquely amenable to imaging among vertebrate models because of its small size, transparency, and ease of anesthesia, making it a useful model to understand host-pathogen interactions. We have performed the first detailed analysis of a viral infection in zebrafish. Infection of zebrafish larvae with a salmonid rhabdovirus adapted to growth at the appropriate temperatures resulted in a predictable succession of pathological signs before death. Detection of infected cells in whole larvae revealed that blood vessels were a major target of the virus, providing an explanation to hemorrhages and subsequent loss of blood flow observed in infected larvae. Destruction of vascular cells caused by the viral infection was readily observed in transgenic larvae with fluorescent endothelium. We could identify the critical moments of the infection with simple temperature shift experiments. This work provides a basis to dissect the role of host factors in controlling the propagation of viral infections.

## Introduction

It is often quite difficult to locate viral infections, as viruses are invisible to the light microscope and are generally noticed by the relatively non-specific symptoms they cause. Specific tools such as monoclonal antibodies allow their detection with techniques that cannot be carried out at the whole-body scale using classical virology models such as rodents. Therefore, important reservoir organs may pass unnoticed and the mechanisms of viral dissemination are hard to establish. The development of systems that allow the detection of viruses in entire animals would help understanding how antiviral treatments or host resistance factors contribute to curb viral infections. They would be especially valuable to assess differential tissue-specific impacts of antiviral responses and treatments.

The zebrafish *Danio rerio* (Hamilton), a well-known model of developmental biologists, is now also turning into a prominent model for the study of host-pathogen interactions [1]. Zebrafish larvae provide a remarkable compromise between ease of imaging, genetic tractability, and homology with human genes and cell types. Their transparency and small size offer a unique possibility to image a whole vertebrate, at medium resolution such that individual cells can be distinguished, or to focus on organ-sized regions where subcellular details can be resolved, using both fluorescence and differential interference contrast (DIC) microscopy. Larvae are easy to anesthetize and can be kept under the microscope for hours or even days. They still lack an adaptative immune response – acquired only at the juvenile stage, by 4–6 weeks of age [2] - but already harbor a powerful innate immune system, with macrophages [3] and neutrophils [4] being the major effector cells. In addition, the zebrafish genome is almost fully known, and overexpression or knockdown of targeted genes in early larvae can readily be achieved *in vivo* by injection at the one-cell stage of synthetic mRNA or antisense morpholino oligonucleotides, respectively. Innate antiviral defenses of teleost fish share many similarities with those of mammals, including the role of interferons as the main orchestrating cytokines [5].

Although no natural virus of the zebrafish is known so far, several viruses from other fish species have been used to experimentally infect zebrafish, including Spring Viraemia of Carp Virus (SVCV) [6], [7], [8], Snakehead Rhabdovirus (SHRV) [9], Infectious Hematopoietic Necrosis Virus (IHNV) [10], [11], Infectious Pancreatic Necrosis Virus (IPNV) [10], Viral Hemorrhagic Septicemia Virus (VHSV) [12], Nervous Necrosis Virus (NNV) [13] and Infectious Spleen and Kidney Virus (ISKNV) [14]. To fully exploit the genetic and optical assets of zebrafish, we are mostly interested in viruses that can infect early larvae within their normal temperature range (22–32°C) and yield highly reproducible clinical signs within a convenient time frame. Zebrafish larvae challenged with SHRV by bath [9] or injected with SVCV [6] or with NNV [13] succumbed readily, within less than 36 hours, making it very difficult to identify conditions that could accelerate the course of infection. Bath challenge with SVCV results in slower kinetics [7] but with high inter-individual variation for onset time of infection signs (JPL, unpublished observations), with only a fraction of fish being infected (as for SHRV), complicating comparisons between treatment groups. In contrast, we found that inoculation of zebrafish with a heat-adapted IHNV resulted in highly reproducible infection courses with convenient kinetics [11], making it the most tractable system to identify the virus target tissues and compare infection spreading in various conditions. We therefore selected it for further analysis.

IHNV is a rhabdovirus first isolated from Pacific salmons in the west coast of North America in the 1950's and later also found in Europe and Asia [15]. It can infect various species of salmonids in the wild, and outbreaks in fish farms represent a significant threat to the salmonid culture industry. In susceptible salmonids, most organs appear to be potential targets of the virus, although according to initial histological examinations, hematopoietic tissues were recognized to be more specifically damaged than others, hence the designation of the virus [16]. Whereas histochemical studies have suggested that leukocytes and endothelium are primary sites of infection [17], the use of a recombinant virus expressing luciferase has revealed the base of the fins to be the entry site of the virus in waterborne-challenged juvenile rainbow trouts [18]. In early experiments performed with adult zebrafish at an unreported temperature, no infection with IHNV could be observed upon bath infection, but intraperitoneal injections resulted in transient viremia and depletion of erythrocyte precursors [10]. However, as IHNV is a cold water virus that normally hardly replicates above 18°C, the tropical zebrafish is not naturally susceptible to the virus; although adult zebrafish may tolerate water temperature below 20°C, larvae do not. We have avoided this problem by using a variant of IHNV that has been adapted to growth at higher temperatures, upon serial *in vitro* passaging on EPC cells at progressively increasing temperatures [11]. The strain used, IHNV25.70 (hereby referred to as IHNV25) can replicate at up to 25°C.

We describe here the outcome of the infection of zebrafish larvae with IHNV25 at 24°C. Prominent clinical signs include slowdown and stop of blood flow, loss of reactivity, hemorrhages and edemas, with death occurring within three to four days. This is accompanied by a continuous rise in virus titer. Patterns of infection in entire larvae could be established using whole-mount *in situ* hybridization (WISH) and whole-mount immunohistochemistry (WIHC). Early virus staining coincided with the major blood vessels. Using transgenic reporter zebrafish, infection and loss of endothelial cells were demonstrated, which may explain much of the observed pathogenesis. By shifting the larvae to a temperature of 28°C, non-permissive for viral replication, we found that a critical threshold resulting in irreversible damage was reached in less than a day, before the first visible clinical signs appeared. This was also found to occur before the onset of a detectable host response in terms of gene expression.

## Results

### Signs of zebrafish larvae infection with IHNV25

**Figure 1:**
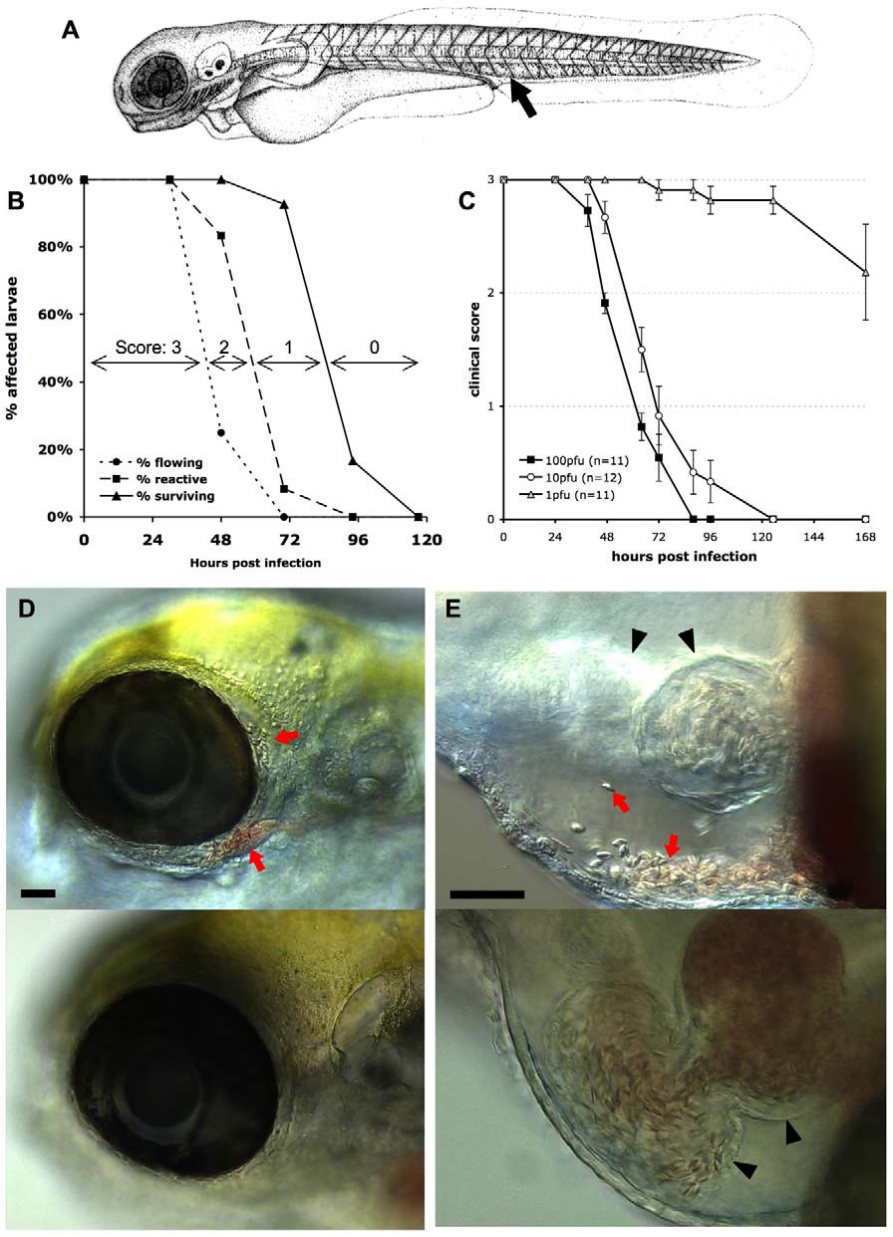
Macroscopic signs of IHNV infection. A. drawing of a 72 hpf larva (length, ∼3 mm) showing the site of inoculation (arrow). B: Onset of disease signs in IHNV-infected larvae. 12 larvae were inoculated at 72 hpf by iv microinjection of ∼100 pfu IHNV25 and incubated individually at 24°C. At regular intervals, they were scored under the dissecting scope for possession or loss of three properties, each shown on a separate curve: active blood flow (circles), reactivity to touch (squares), or survival (triangles). All control uninfected larvae (*n* = 6) remained reactive with strong blood flow during the whole time interval. Experiment repeated three times with comparable results. C. Effect of the dose of injected virus on disease course, using a clinical scoring method. Larvae (77 hpf) were injected with varying doses of IHNV25, incubated individually at 24°C, and regularly checked for survival, blood flow in the tail, and reactivity to touch, to attribute a clinical score to each individual (criteria illustrated on panel B; see Methods for details). Graph displays mean clinical score for each group; error bars correspond to standard error of the mean (s.e.m). Experiment repeated twice with similar outcome. D and E: Accumulation of erythrocytes in larvae 40 hours after injection of IHNV25. Lateral views, anterior to the left, dorsal to the top; live DIC imaging. Top panel: IHNV-infected larva; bottom panel, uninfected control. C (10x objective) head with erythrocytes accumulated around the eye (arrows). D (20x objective) pericardial cavity. Arrows, erythrocytes. Arrowheads, heart (left arrowhead: ventricle, right arrowhead, atrium. Because different focal planes have been selected to provide the best view of the pericardial cavity, the ventricle is out of focus for the infected larva). Scale bars, 50 µm.

We previously reported that zebrafish larvae were susceptible to intravenous (iv) infection with the IHNV25 strain when incubated at 24°C [11]. We studied the clinical signs of infection in more detail. Zebrafish larvae aged ∼72 hours post fertilization (hpf) were injected iv with 60 to 120 plaque forming units (pfu) of IHNV25 in the enlarged venous plexus located posteriorly to the urogenital opening (Figure 1A). They were then incubated individually in 24-well plates at 24°C and monitored at regular intervals under the dissecting scope. During the first 30 hours post infection (hpi), larvae appeared to be clinically healthy. Then the blood flow started to slow down, until it stopped completely, often first in the tail, then in the entire body, by approximately 48 hpi (Figure 1B). This qualitative observation could be confirmed by quantitative assessment of blood speed (Figure S1 in Text S1). This was not due to an arrest of the heart, which continued to beat, although more weakly. Larvae then gradually lost reactivity, with progressively weaker reaction upon gentle pricking, until they became completely inert. Death - defined by complete absence of movement, including any residual heartbeat, and readily followed by decomposition - occurred for all the fish, generally between 65 and 96 hpi.

For subsequent experiments, and to facilitate future studies that will assay *in vivo* susceptibility to IHNV, we defined a simple clinical score from 0 to 3, based on these signs of infection that can be easily scored without anesthesia of larvae: loss of blood flow in the tail, loss of reactivity to touch, and death (see material and methods). As illustrated on Figure 1B, these criteria define three progressive steps of pathogenesis which were translated into scores providing richer information than endpoint mortality.

Inoculations of varying doses of virus yielded a clear dose-dependent response (Figure 1C); however, even if kinetics were slowed down at lower doses, signs occurred in a similar order. The 50% lethal dose over 7 days was quite low, inferior to 10 pfu. The dose of 100 pfu, however, was kept as the standard inoculum for further experiments, because it yields less inter-individual variation.

Other signs of infection were frequently observed, although not as consistently as the previous ones. When blood started slowing down, accumulation of erythrocytes could often be observed in various places, such as around an eye (Figure 1D), in the duct of Cuvier just upstream of the heart, on the top of the head, or in the caudal venous plexus (not shown). Although it was difficult to discern whether this resulted from vessel leakage or from accumulation inside a vessel, some cases clearly resulted from hemorrhage, as when erythrocytes were observed inside the pericardial cavity (Figure 1E). Skin damage was also frequently seen, especially along the lateral midline of the trunk and on the ventral side of the yolk ball. Edemas were also commonly observed, affecting mostly the pericardial cavity and the head. At late stages, necrotic foci were often visible in the brain (not shown).

### Course of infection in the whole fish

In order to better understand the course of IHNV infection in this system, we assessed the spread of the virus over time by different quantitative methods.

**Figure 2:**
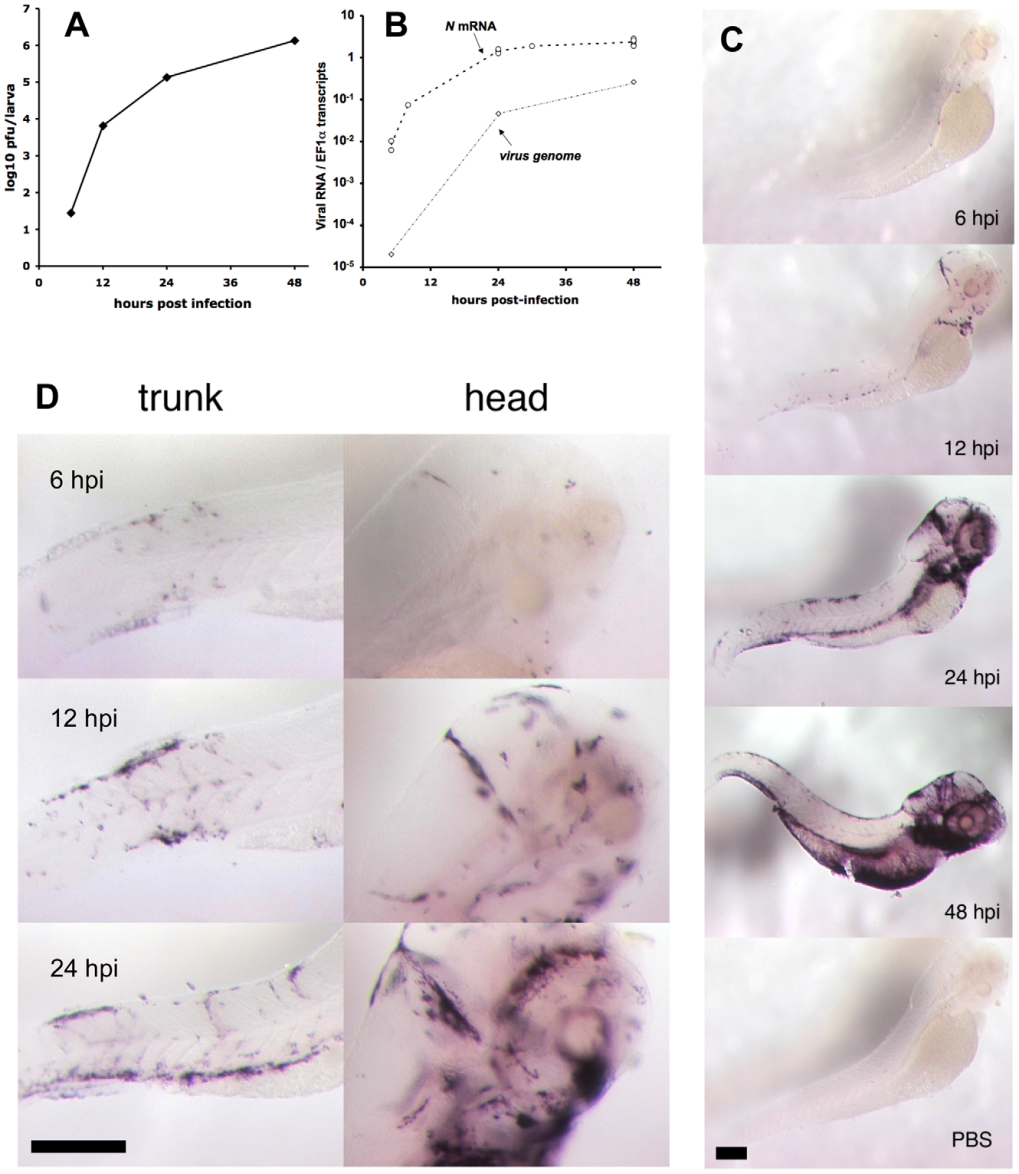
Progress of IHNV infection. A. Quantification of viremia over time. Larvae were inoculated with IHNV as in Figure 1B. At regular intervals, pools of five larvae were snap-frozen, and virus titers measured on a layer of EPC cells. Titers were always below the threshold of detection (5 pfu) in uninfected control larvae. Experiment repeated twice with similar results. B. Assessment of viremia in whole larvae using qRT-PCR. Quantification of *N-IHNV* transcripts (circles) or viral genomes (diamonds) over housekeeping gene *EF1α* transcripts. For *N-IHNV* transcripts, data pooled from five experiments; dashed line join mean values. Viral RNAs were undetectable in control larvae. C. Detection of infected cells using WISH with a *N-IHNV*-specific probe. Representative images of PTU-treated larvae inoculated as in Figure 1B (or injected iv with PBS for bottom image) and fixed at the indicated times post-infection (24 hpi for PBS control). Magnification: 3.2x. D. details of ISH images at higher (10x) magnification. Scale bars, 200 µm.

Firstly, the numbers of infectious virions were measured by plaque assays on monolayers of EPC cells. A few dozens particles were detectable by 6 hpi; then, near-exponential growth was found (Figure 2A), providing definitive evidence for functional viral replication in zebrafish. At 48 hpi, up to one million pfu per larva were found. Since a 5dpf zebrafish larva weighs about a 0.5 mg [19], this translates to viral concentrations in the order of 10^9^ pfu/g. Secondly, we measured the expression of a viral mRNA transcript by qRT-PCR on whole larvae, choosing the *N* gene as the most highly expressed viral gene [20]. Increases of *N-IHNV* gene expression paralleled the rise of viral titers (Figure 2B). Thirdly, we quantified viral negative and positive genomic strands (genome plus antigenome) by qRT-PCR; predictably, levels were lower than for the *N* transcript but progression was comparable (Figure 2B).

Finally, the spatial distribution of infected cells was determined by WISH with a probe complementary to the *N* gene. Stained cells could be detected in infected larvae at least as early as 6 hpi (Figure 2C), and their number increased then steadily over time, in accordance with our previous quantifications. The distribution of infected cells was variable from larva to larva, but some common patterns were observed. Early infection was almost systematically detected at the location of the main veins at this developmental stage [21]: in the tail, the cardinal vein; in the head, the dorsal longitudinal vein, posterior cerebral vein, primary head sinus, and inner optic circle; in the anterior ventral region, the duct of Cuvier. A few infected cells were also systematically detected in the heart. Observation of larvae fixed at 12–24 hpi points revealed a more intense staining in all these areas; examination of stained larvae at higher magnification suggested a spread from endothelium to adjacent cell layers (Figure 2D). At 48 hpi the strongest staining was systematically observed in the branchial arches (Figure 2C).

### Vascular endothelium is a primary target of IHNV

WISH patterns suggested that the vascular endothelium was a primary target of IHNV. To ascertain this we infected *fli1:GFP* transgenic larvae, which express GFP in all endothelial cells [22]. Instead of WISH, which destroys GFP fluorescence, the distribution of virus-infected cells was analyzed by whole-mount immunohistochemistry (WIHC), using the 4B3 and 19B7 monoclonal antibodies (mAb) directed against the P and G protein of IHNV, respectively. This technique was found to be less sensitive than WISH due to a higher background, especially with 4B3 (Figure S2 in Text S1), but the general staining patterns were similar, supporting the specificity of our labelings.

**Figure 3:**
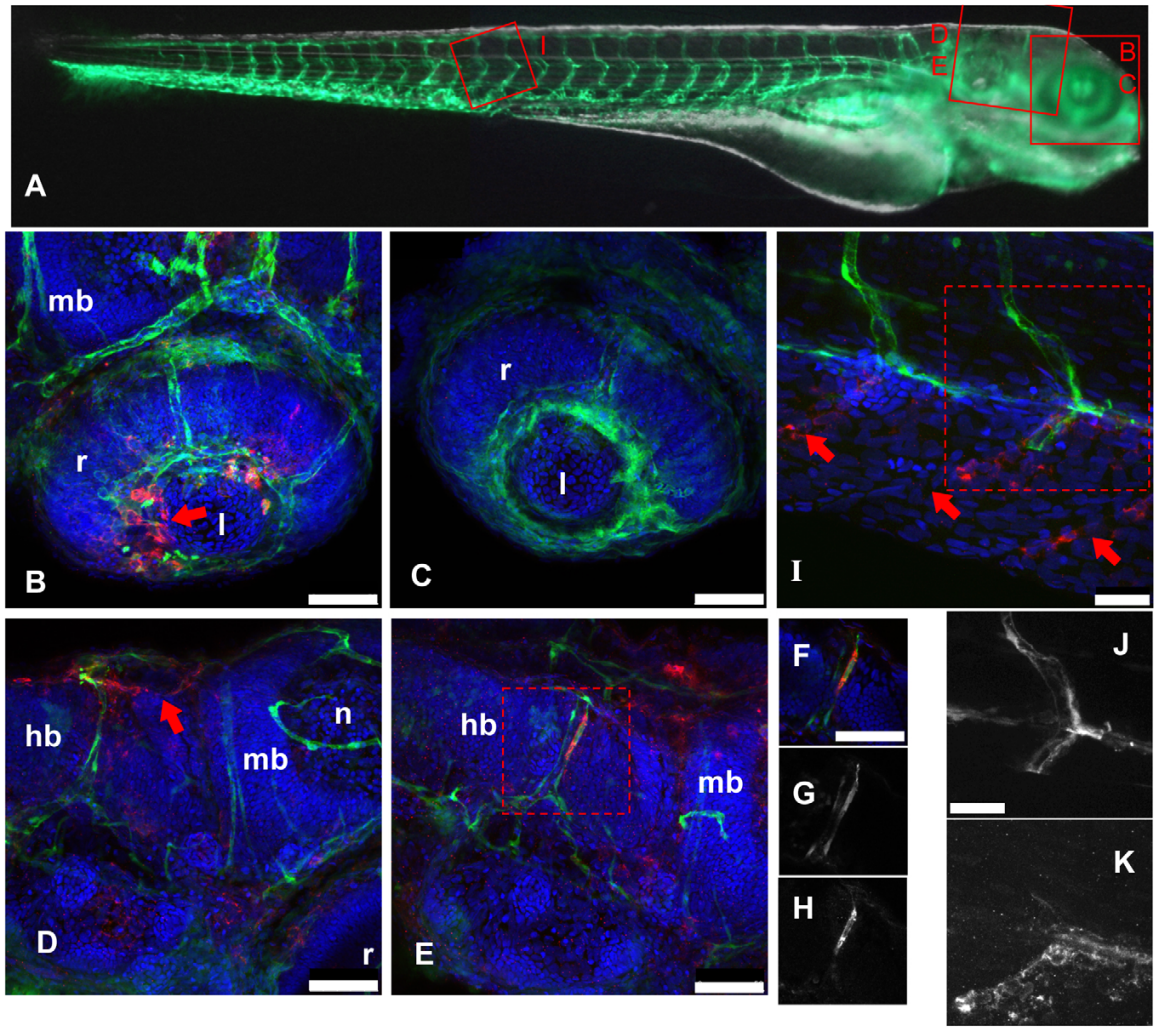
IHNV infects and disrupts blood vessels. A: scheme of the imaged regions (corresponding panel indicated with red letters), overlaid on a composite transmission (grayscale) and GFP fluorescence (green) picture from a 4dpf *fli1:GFP* larva viewed laterally (images taken with a stereomicroscope; to get a more readable GFP signal, short exposure of the head has been combined with longer exposure of the rest of the body). B–K, confocal images (maximal projections from multiple Z-stacks) of WIHC stainings of *fli1:GFP* larvae fixed 24 hpi after IHNV inoculation at 72 hpf (except C, uninfected control larva); latero-dorsal views, anterior to right, dorsal to top. Colours in B–F and I: Red: IHNV P protein; green: GFP; blue: nuclei. B: eye, showing disruption of the inner optic circle, with infected cells located close to the damaged site (arrow; compare with C). D and E: brain region of two infected larvae, showing variable pattern. Notice in D the infection and loss of GFP expression of dorsal longitudinal vein (arrow). E shows infection of a cell of the posterior cerebral vein; to demonstrate colocalization of GFP and viral protein, single Z-plane images (cropped to the boxed area in E) are shown in F–H with overlaid signals in F, GFP signal in G, and P-IHNV staining in H. I: view of intersomitic vessels, showing infection and loss of GFP of ventral part of these vessels (arrows indicate where endothelial cells are expected to be found) while the dorsal part appears intact. Partial colocalization of GFP and P-IHNV in the ventral half of one vessel is shown in J and K (GFP and P-IHNV signal channels, respectively). B–H, 40x objective; scale bar, 50 µm. I–K 63x objective; scale bar, 25 µm. hb: hindbrain; l: lens; mb: midbrain; n: neuropile; r: retina.

*fli1:GFP* larvae were infected with IHNV25, fixed at 24 hpi, processed for WIHC with the 4B3 mAb, and analyzed by fluorescence confocal microscopy (Figure 3). Most cells stained with the antibody (in red) were found at a position close to the expected location of a vessel. Co-localization with GFP (in green) was relatively rare, and vessels generally appeared to be disrupted near places of viral P protein expression (Figure 3B–D). Nevertheless, in some cells, unambiguous co-localization of GFP (in both cytoplasm and nucleus) and P-IHNV (in cytoplasm only) was observed (Figure 3E–H). This suggested that infection of an endothelial cell with IHNV quickly resulted in the death of the cell, or at least loss of GFP expression. This was further strengthened by observations of some infected intersomitic vessels (Figure 3I–K): the dorsal half of one vessel was found to be virus free and expressing GFP, while no GFP expression was found on the ventral-most third, where, in contrast, cells stained for P-IHNV were found at the expected vessel location. One doubly-labeled cell is visible just below the midline. Again, frequent staining of cells just next to the vessel location suggested that the infection spread from the endothelial cells to underlying tissue.

Observations of P-IHNV expressing cells in the heart were generally more consistent with infection of isolated myocardial cells rather than endocardial cells (not shown).

The anti-G mAb allowed us to detect infected cells as early as 10 hpi (but not at 6 hpi), and we used it to conduct a time-course IHC analysis from 10 to 24 hpi. The number of infected cells was observed to increase steadily over time, and the majority of infected cells were found at places where endothelial cells are expected, often (but not always) expressing GFP (Figure S3 in Text S1). Some infected cells were located outside of the vessels, but this was less frequent at early time points.

In conclusion, these observations establish that some vascular endothelial cells are infected with IHNV prior to the appearance of clinical signs. They also suggest that this rapidly results in disruption of blood vessels and dissemination of the virus to neighboring cells.

### IHNV affects endothelial cells but not erythrocytes

**Figure 4:**
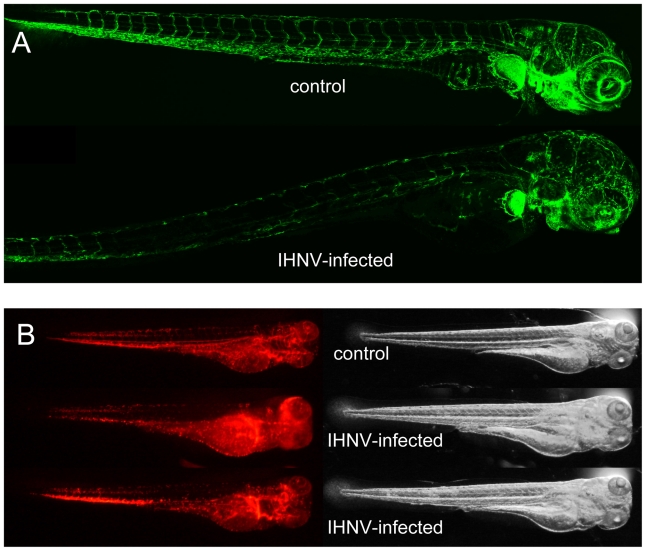
IHNV infection destroys vascular endothelium but not erythrocytes. A. Confocal images of representative *fli1:GFP* fish at 49 hours post inoculation; maximal projection from Z-stacks and mosaic reconstruction. Because of individual variation in GFP expression among *fli1:GFP* larvae, fluorescence levels have been globally adjusted (here, increased in the IHNV-infected fish) to match in the non-endothelial cells of the pectoral fin that also express GFP. B. Fluorescence (left) and transmission (right) images of *gata1:DsRed* larvae at 48 hpi, taken with a fluorescence stereomicroscope; exposure settings kept identical for all animals. The two bottom animals have been selected to illustrate the variable distribution of red blood cells immobilized in infected larvae.

The overall loss of GFP expression by endothelial cells in *fli1:GFP* larvae could be readily observed in live imaged animals; from 48 hpi the difference between infected and control larvae was striking (Figure 4A). Erythrocytes (which are nucleated in zebrafish) and their precursors were also likely targets of the virus; anemia has been described after IHNV injection to adult zebrafish [10] and these cells are as exposed as endothelial cells with iv inoculation. To test this, we infected and live imaged *gata1:DsRed* reporter larvae, in which DsRed is expressed in erythrocytes and their precursors [23]. The distribution of DsRed-expressing cells was different between control and infected animals and consistent with accumulation of red blood cells in various spots due to loss of blood flow; however, the overall level of DsRed expression did not appear to be decreased at 48 hpi (Figure 4B), indicating that in contrast to endothelial cells, erythrocytes are spared during IHNV infection. This conclusion was further strenghtened by careful examination of *fli1:GFP* larvae stained with the 4B3 or 19B7 mAbs (see above), where no viral P or G protein expression could be detected in cells inside the lumen of blood vessels (Figure 3 and not shown).

### Temperature shift identifies the irreversible point in pathology

**Figure 5:**
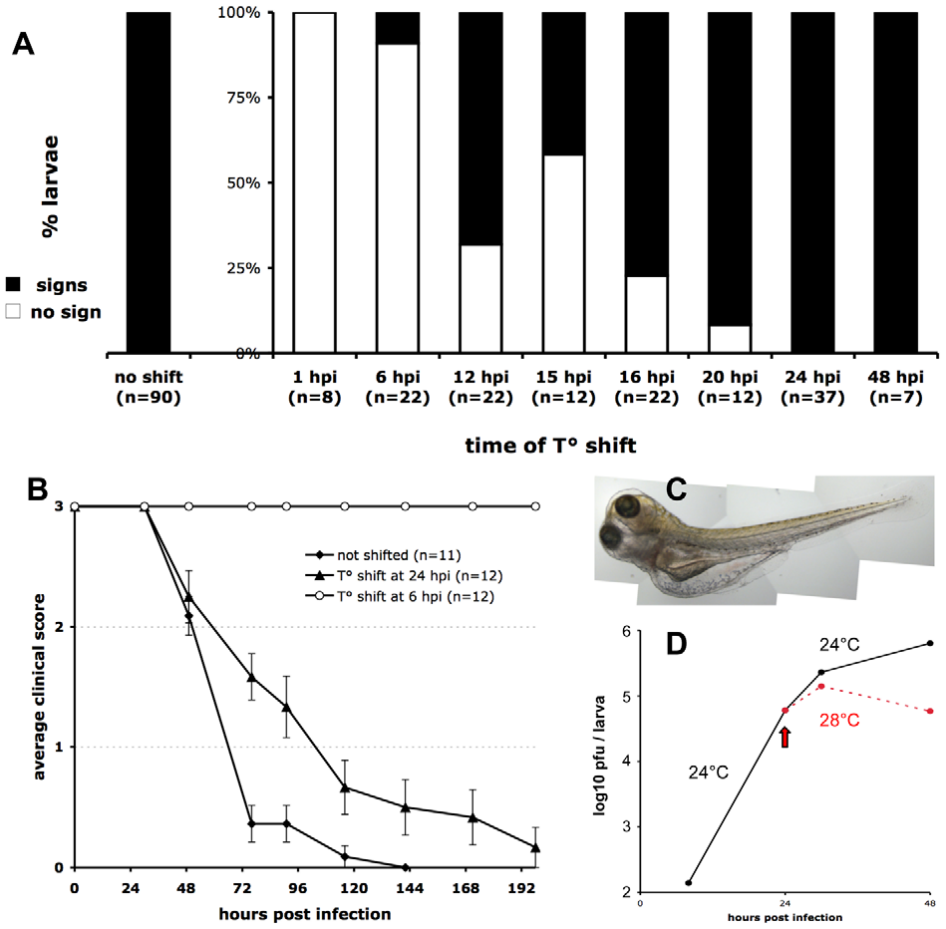
Controlling the progression of IHNV infection by temperature shift. A. Early temperature shifts impairs development of signs of infection in zebrafish larvae inoculated with IHNV25. Larvae (65 to 75 hpf) were infected with 65 to 100 pfu of IHNV25, transferred in individual wells, incubated at 24°C, then shifted to 28°C at the indicated time. They were checked regularly for signs of infection (loss of blood flow or reactivity, gross edemas, death). Larvae that did not develop any sign up to 7 days post-infection were scored as « no sign » (open bars). Results pooled from 8 separate experiments. B. Representative time-course of onset of disease signs in temperature-shifted larvae, in one of the experiments included in A. Clinical scores measured as for Figure 1C; error bars represent s.e.m. C. Photograph of an infected larvae shifted to 28°C at 24 hpi, taken at 72 hpi, showing typical generalized edema. D. Comparison of infectious viral titers in IHNV-infected larvae kept at 24°C (black full line) or shifted to 28°C at 24 hpi (dashed red line). Experiment repeated twice with similar results.

We took advantage of the fact that the IHNV25 strain does not grow above 25°C to stop the progression of infection in zebrafish larvae at any stage, with simple temperature shift experiments. First, we ensured that either growth or cytopathic effect of IHNV25 can be observed *in vitro* at 24°C but not at 28°C (Figure S4 in Text S1). Larvae were infected iv as previously, incubated at 24°C for a certain time, and then shifted to the non-permissive temperature of 28°C. During the entire course of the experiment, individual larvae were regularly observed for occurrence of symptoms (Figure 5A). If the shift was performed immediately, no pathology occurred. When the shift was performed at 6 hpi, no subsequent signs of infection could be detected in almost all larvae. In contrast, if the shift was performed at 24 hpi or later, irreversible pathogenesis ensued for all larvae. The critical turning point in the infection, with approximately 50% of the larvae being rescued without any of the above-described clinical signs, was 12 to 15 hpi.

Before dying from the infection, temperature-shifted larvae displayed the same signs as described previously for unshifted larvae, in the same order, but delayed (Figure 5B); in addition, they exhibited edemas of impressive proportions (Figure 5C).

Quantification of IHNV titers or *N-IHNV* transcripts in T°-shifted larvae revealed, as expected, that infection was reduced as compared to non-shifted larvae (Figure 5D). However, high amounts of virus were still detectable, indicating that T° shift to 28°C did not destroy the virus and suggesting it did not rescue already infected cells. The T° shift probably prevented infection of new cells, interfering with either viral entry, replication or assembly.

### Host response arises too late during infection

**Figure 6:**
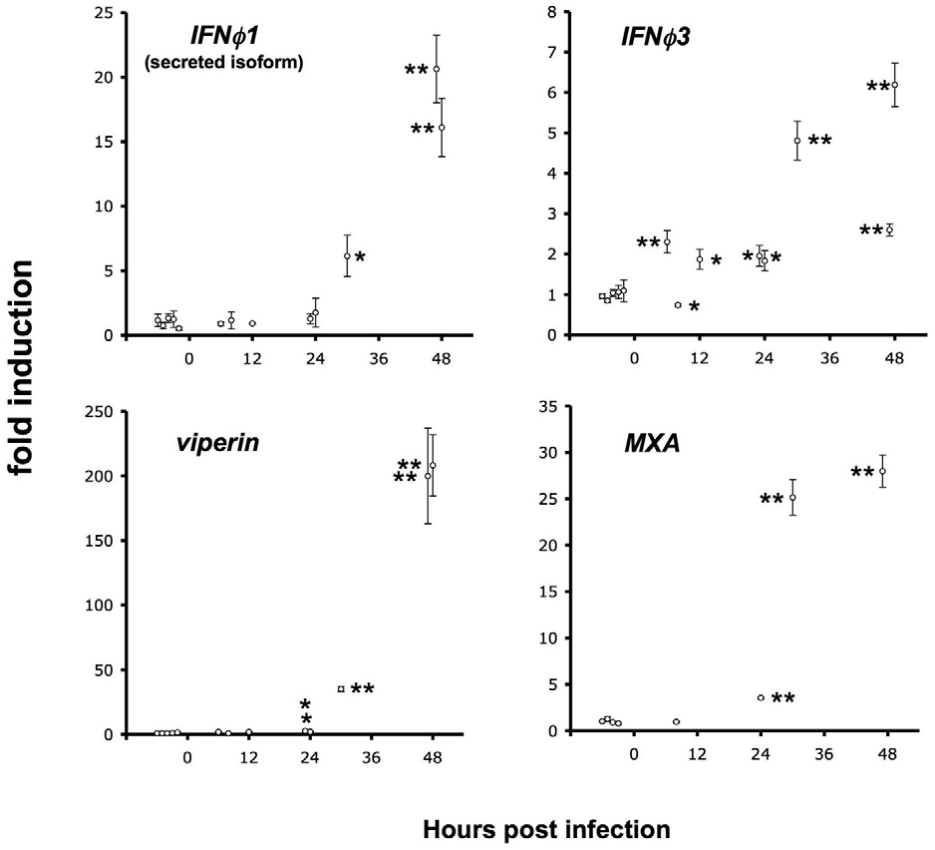
Late induction of host response. qRT-PCR measurement of the expression of IFN genes (top panels; left, splice isoform of *ifnϕ1* corresponding to the inducible, secreted cytokine; right, *ifnϕ3*) and of IFN-induced genes (bottom panels; left, *viperin/rsad2*, right, *MXA*). Assays performed on RNA extracted from entire larvae, either infected with ∼100 pfu of IHNV and incubated at 24°C for the indicated time, or uninfected controls (five or four samples, displayed before the “0 hpi” mark). Measured values normalized to the mean of the control set. Error bars, SD of triplicate qPCR measures. Significant difference from control set displayed as ** (*p*<0.0001) or * (*p*<0.01); unpaired *t*-test.

We have previously shown [11] that larvae can be partially protected from IHNV infection by injection of recombinant interferon (IFN) a few hours before viral challenge and that expression of both *ifnϕ1* and *ifnϕ3* is induced at 48hpi by IHNV infection. Both interferons induce the expression of many genes including *viperin* (also known as *vig1* or *rsad2*) and *MXA*. To analyse the kinetics of expression of host antiviral genes during the course of the infection, we measured by qRT-PCR the expression of *ifnϕ1*, *ifnϕ3*, and *viperin* at 6, 8, 12, 24, 30 and 48 hpi (Figure 6). Potent induction of all these genes could only be detected at 30 or 48 hpi. Stastically significant, but very weak (less than 4-fold) induction of *viperin* or *MXA* was detectable at 24 hpi. Levels of I*FNϕ3* were sometimes slightly elevated (about 2-fold) early after infection. As T°-shift experiments have shown that IHNV infection causes irreversible damage before 24 hpi, this result strongly suggests that the endogenous host response comes too late to exert any significant control over the viral infection.

### IFN signalling does not cause disease signs

Even if overdue, the host IFN response may be responsible for some of the disease signs, which are also observed after the point of no return. To test this, we injected 72 hpf *fli1:GFP* larvae with recombinant IFN, and carefully monitored them afterwards. We tested both groups of fish IFNs by using either 100 pg of IFNϕ1 or 1 ng of IFNϕ2, doses that result in strong *viperin* induction 6 hours post-injection, and to significant resistance to a challenge with IHNV25 when compared with BSA-injected controls ([11] and data not shown). We observed the larvae injected with IFN, but not challenged with virus, for any sign linked to the disease, such as mortality, loss of reactivity, decrease of blood flow, haemorrhages, or edemas, and found no difference with control larvae. We also imaged them by fluorescence microscopy at 6, 24 and 48 hours post-injection of the cytokine, and did not observe any dampening of the GFP signal in endothelium (data not shown). These negative results were obtained in two independent experiments. We conclude that disease signs are unlikely to be caused by the host IFN response, but probably reflect virus-caused damage.

## Discussion

We describe in this paper the spread of a viral infection throughout an entire organism, something that, to our knowledge, has not been done before in a vertebrate. Taking advantage of the small size and optical accessibility of zebrafish larvae, it is possible to identify infected cells anywhere in the body. This provides us with a very useful model to study the effects of antiviral drugs or of the various elements of the host response. A particular advantage of this system over other approaches resides with the possibility to directly compare organ specificity. Many viruses are known to take up residence in reservoir organs where they are harder to detect and are less exposed to drugs and/or immune responses, such as the central nervous system [24], [25].

Although the approaches we developed here could be readily applied to other viruses using the adequate probes or antibodies, the heat-adapted IHNV virus has specific advantages. First, gross clinical signs of infection are easy to follow, reproducible, and occur in a highly synchronous fashion in groups of infected larvae (Figure 1), making it simple to compare experimental situations. The kinetics of the infection is convenient: death does not occur in the precipitous ways seen with other viruses such as SVCV [6] -making it possible to identify factors that would accelerate infection- yet signs occur early enough to allow the use of synthetic mRNA and morpholino-based gene gain- and loss-of-function approaches available in zebrafish larvae. Moreover, using very simple temperature shift experiments, it is possible to manipulate the replication of the virus in this poikilothermic animal (Figure 5). Finally, this virus is amenable to reverse genetics.

This system also has some significant drawbacks. IHNV is not a natural pathogen of zebrafish - however none has been characterized so far. The virus has to be microinjected to establish infection, precluding the study of events involved in virus entry. Nevertheless, it affords a reliable system to study the subsequent systemic spread of the virus. Although some other viruses can enter zebrafish larvae by more natural routes, they do not result in the highly predictable infection course obtained with this IHNV strain, and are therefore less amenable to experimental manipulation.

Our observations highlight vascular endothelial cells as the primary targets in the pathology of this experimental infection. The first cells expressing viral genes are found where major veins are localized (Figure 2); infected endothelial cells could be observed (Figure 3). As the infection progresses, the vessels become disrupted and other cells are found infected next to the former location of the missing endothelial cells, suggesting that the virus spreads to the neighboring tissues mostly by cell-to-cell contact, even though direct translocation of bloodborne virus to these tissues through fenestrated endothelium cannot be ruled out. Disruption of vessel integrity can explain much of the observable signs of disease, such as slowing down and arrest of blood flow despite continuing heartbeat, hemorrhages, and edemas. There is probably a threshold level of damage to the endothelia, below which the larvae can still maintain or regenerate vessel integrity, as suggested by temperature shift experiments: many larvae could fully recover when shifted to the non-permissive temperature before 18 hpi, despite the fact that infected vessels were revealed by WISH in all animals at 6 hpi.

There seems to be a preferential infection of veins over arteries, at least in the earlier phases of the infection; this may be explained by the higher endocytic capacity of zebrafish venous endothelial cells (PH, unpublished data) reflecting a general property of certain subsets of endothelial cells in all vertebrates [26].

Are there other cells types infected by IHNV, and what is their contribution to the pathology? Our observations of *gata1:DsRed* transgenic larvae indicate that erythrocytes and their precursors are not early targets of the virus (Figure 4). Our preliminary, unpublished observations also suggest that neither neutrophils nor thrombocytes are targeted by the virus. The apparent tropism of the virus for endothelium among blood-exposed cells clearly deserves more investigation. Fibronectin has been shown to act as a primary receptor for IHNV in trout cells [27], and further experiments performed *in vitro* with zebrafish cells have pointed out that IHNV entry is mediated by a minor truncated isoform of fibronectin (FN2) located at cell surfaces [28]. It would be interesting to check whether this isoform is specifically expressed by vascular endothelial cells. Unfortunately, almost all of the sequence of the FN2-encoding transcript is also included in the transcript encoding the main fibronectin isoform, precluding WISH analysis. We also plan to study in more detail the infection of non-hematopoietic tissues; notably, it will be of particular interest to establish how the virus propagates in the brain (in which necrosis is often observed at late stages) from infected brain vasculature, as it exemplifies one of the strategies that allow pathogens to cross the blood-brain-barrier.

As shown here, zebrafish larvae are unable to mount a detectable interferon response before irreversible damage has been caused by the infection (Figure 6). This is clearly not the consequence of an immature state of the immune system, as a response to SVCV could be detected much earlier [6]. We suspect that, like other rhabodviruses [29], IHNV has the ability to hinder or delay the induction of the host interferon response. Identifying the precise molecular mechanisms at play will be an important goal in the future.

In conclusion, the IHNV/zebrafish model we have established constitutes the first example of a system where a viral infection can be imaged in an entire vertebrate host. Our observations suggest the following scenario of viral dissemination: first, via an hematogenous route, leading to infection of vascular endothelial cells throughout the body. The ensuing destruction of endothelial cells disrupts blood flow, causing hemorrhages and edemas. The infection of a sufficient number of vascular cells is probably sufficient to yield irreversible damage. However, it also results in a second mode of infection, affecting underlying tissue via cell-to-cell or very short distance virus transfer. This sequence of events is likely to hold true for a number of human viruses causing hemorrhaging diseases. The validity of this hypothetical scenario is testable, as it predicts that experimental manipulations that result in overexpression of genes with antiviral activity (i.e. appropriate IFN-stimulated genes) specifically in vascular endothelial cells should result in efficient protection of the host with reduced side effects compared to ubiquitous overexpression. Thanks to the already available genetic tools, endothelium-specific inducible expression would be relatively easy to achieve in zebrafish; the endocytic properties of veinous vascular cells may also be exploited to target drugs to these cells, and this could be monitored in real time. Combined with the assessment of organ-specific distribution of virus in the organism, such studies have the potential to help designing more targeted, safer treatment regimens for human viral diseases.

## Material and Methods

### Ethic statement

All the animal experiments described in the present study were conducted at the Institut Pasteur according to the European Union guidelines for the handling of laboratory animals (http://ec.europa.eu/environment/chemicals/lab_animals/home_en.htm) and were approved by the Institut Pasteur animal care and use committee and by Direction Sanitaire et Vétérinaire de Paris under permit #A-75-12-22.

### Fish

Wild-type AB, initially purchased from the ZIRC (Zebrafish International Resource Center, Eugene, OR), transgenic *fli1:GFP* [22], and transgenic *gata1:DsRed* [23] were raised in our fish facility. Eggs were obtained by marble-induced spawning, bleached according to protocols described in The Zebrafish Book [30], and then kept in petri dishes containing Volvic source water supplemented with 0.3 µg/ml of methylene blue. Depending on the desired speed of development, embryos were raised at 28°C or 24°C before infection; all staging in the text refers to the standard 28,5°C developmental time. A few hours before injection, embryos were dechorionated manually. Larvae were anesthetized with 200 µg/ml tricaine (Sigma-Aldrich).

### Virus

Generation of the IHNV25.70 strain has been described in [11]. The virus was propagated on EPC cells (ATCC# CRL-2872). Virus-containing cell culture supernatants were aliquoted and stored at −80°C until use. Just before injection, the virus was diluted (if necessary) to the appropriate concentration with PBS containing 0.1% phenol red.

### Infection and clinical score

Larvae were infected by iv microinjection in the caudal vein or aorta as described in [31]. Infected larvae were then distributed in individual wells of 24-well culture plates, containing 1 ml of Volvic water each. Larvae were regularly observed with a stereomicroscope to check for the appearance of clinical signs of infection.

A clinical score from 0 to 3 was determined without anesthesizing the larvae by checking for movement of blood cells in the tail and by gently pricking the side of the head with a soft paintbrush. Larvae with visible blood flow in the tail - precisely, in any vessel located posterior to the urogenital opening -, which were always reactive, were attributed a score of 3. Larvae with blood arrested in the tail (even if flowing elsewhere) but that still swam away (at least one body length of distance) when pricked were given a score of 2. Larvae that did not swim away after three pricking attempts, but still had any detectable heart beat when oriented on their side were assigned a score of 1. Larvae with no movement whatsoever were considered dead with a score of zero.

Scoring has been performed in a blind fashion at least once for each type of test.

### Imaging

Imaging was performed as described in detail in [31]. Briefly, video-enhanced DIC images of live larvae were taken using a Nikon Eclipse 90i microscope equipped with a Hitachi HV-C20 camera and movies captured on miniDV tapes; single frames were later captured using the BTVpro software. Images of larvae stained by WISH were taken with a Leica MZ16 stereomicroscope using illumination from above. Images of whole live larvae were taken with a similar stereomicroscope fitted with a Nikon DS-5Mc camera, using oblique illumination. Confocal images of live or fixed larvae were taken with a Leica SPE inverted confocal microscope. Images were processed with the LAS-AF (Leica), ImageJ and Adobe Photoshop softwares.

### Plaque assay

Titers of infectious virions were measured by plaque assay on monolayers of EPC cells. Larvae to be assessed were anesthetized with tricaine, transferred as groups of 5 larvae to a microtitration tube with no more than 30 µl of water, snap-frozen on a bed of dry ice and stored at −80°C until processing. Larvae were homogeneized by grinding them with a pestle fitted to the tubes, and 100 µl of culture medium supplemented with 2% FCS was added. Supernatants were cleared by a 5 min centrifugation at 930 g, and serially diluted in duplicates for the plaque assay. The infection was performed at 24°C under a layer of methylcellulose (0.75% final concentration) for three days after an adsorption step at 14°C for one hour in liquid phase. The plaques were then counted after treatment by formaldehyde (10%) and staining using crystal violet (1% final dilution).

### qRT-PCR

RNA was extracted from snap-frozen larvae using Trizol (Invitrogen). cDNA was obtained using M-MLV H- reverse-transcriptase (Promega) with a dT_17_ primer, except for IHNV genome quantification where a random N_10_ primer was used. Quantitative PCR was then performed on an ABI7300 thermocycler (Applied Biosystems) using SYBR green reaction power mix (Applied Biosystems). The following pairs of primers were used:

*EF1α*: GCTGATCGTTGGAGTCAACA and ACAGACTTGACCTCAGTGGT

*N-IHNV*: CACTGGACTCAGAGACATCA and CTGCAAGCTTGTTGTTGGGA

IHNV genome: CACTGGGTGGAATTCCCTTT (in *G* gene) and CAATACTCGCTGCATCCTCT (in *NV* gene)

*IFNϕ1*: TGAGAACTCAAATGTGGACCT and GTCCTCCACCTTTGACTTGT

*IFNϕ3*: GAGGATCAGGTTACTGGTGT and GTTCATGATGCATGTGCTGTA

*viperin*: GCTGAAAGAAGCAGGAATGG and AAACACTGGAAGACCTTCCAA

*MXA*: GACCGTCTCTGATGTGGTTA and GCATGCTTTAGACTCTGGCT

Quantifications were performed on triplicate wells, and taking into account the previously measured yield of the reaction as described in [32]. To normalize cDNA amounts, we have used the housekeeping gene *EF1α* transcripts, chosen for its high and very stable expression from 12 to 120 hpf [33]; error bars represent standard deviation of the measured ratios.

### In situ hybridization

WISH was performed using standard protocols [30]. To generate the probe, the full-length coding sequence of *N-IHNV* [34] was subcloned in antisense orientation in the pCS2+ vector, which was then linearized with NotI and transcribed *in vitro* with SP6 polymerase (Promega). The signal was very high and revealed in five to ten minutes.

### Immunohistochemistry

WIHC was performed as described [35] using, as a primary antibody, either the 4B3 mAb antibody specific of IHNV P (phosphoprotein) or the 19B7 mAb antibody specific of IHNV G (glycoprotein) [36] diluted 1/500^th^; and, as a secondary antibody, Cy3-labelled goat anti-mouse antibody (Jackson Immunoresearch) diluted 1/300^th^. Larvae were counterstained for 45 min at room temperature with 2 µg/ml Hoechst 33342 (Molecular Probes) before being progressively transferred to 80% glycerol.

### Accession numbers of genes studied in this article

zebrafish genes or proteins:

EF1α: NM_131263; IFNϕ1: NM_207640; IFNϕ2: NP_001104552; IFNϕ3: NM_001111083; viperin: NM_001025556; MXA: NM_182942

IHNV genes or proteins:

Genome: NC_001652; N: NP_042676; P: NP_042677

## Supporting Information

Text S1 Supplementary Material and Methods, Supplementary References, and Supplementary Figures S1-S4.
(3.09 MB PDF)
