# Supplementary material for: Whole-Body Analysis of a Viral Infection: Vascular Endothelium is a Primary Target of Infectious Hematopoietic Necrosis Virus in Zebrafish Larvae
Source: PLoS Pathog. 2011 Feb 3;7(2):e1001269. doi: 10.1371/journal.ppat.1001269 (PMC3033377; doi:10.1371/journal.ppat.1001269)
Supplement: Text S1 — Supplementary Material and Methods, Supplementary References, and Supplementary Figures S1-S4. (3.09 MB PDF) [file ppat.1001269.s001.pdf]

Supplementary information to Ludwig et al., " Whole-Body Analysis of a Viral Infection: Vascular Endothelium is a Primary Target of Infectious Hematopoietic Necrosis Virus in Zebrafish Larvae"

Supplementary Material and Methods, Supplementary References, and Supplementary Figures S1-S4

Supplementary Material and Methods

Fish

Transgenic *CD41:GFP* fish [1] were raised in our fish facility and larvae were obtained as for the other strains.

Virus

The wild-type IHNV (strain 3287) used for comparison with IHNV25 has been described in [2] and propagated on EPC cells.

Blood cell speed

Blood speed was determined by measuring the speed of green fluorescent thrombocytes in the dorsal aorta of *CD41:GFP* transgenic larvae. Briefly, larvae were anesthetized with 160 µg/ml tricaine, then oriented and immobilized in 1% low melting point agarose. Before and during imaging, larvae were maintained at 24°C. Green fluorescence images were taken approximately every 350 milliseconds with a Leica MacroFluo fluorescence stereomicroscope. Individual thrombocytes were tracked manually on series of images; distances travelled were measured using the MetaVue imaging software. For each cell, the first and last images in the series with the cell visible in the aorta were selected and speed (µm/s) was calculated as the quotient between the distance in micrometers travelled by the cell and the duration of the acquisition interval between these two images.

Supplementary references

1. Lin HF, Traver D, Zhu H, Dooley K, Paw BH, et al. (2005) Analysis of thrombocyte development in CD41-GFP transgenic zebrafish. Blood 106: 3803-3810.

2. Hattenberger-Baudouy A-M, Danton M, Merle G, Torchy C, de Kinkelin P (1989) Serological evidence of infectious hematopoietic necrosis in rainbow trout from a French outbreak of disease. J Aquatic Anim Health 1: 126-134.

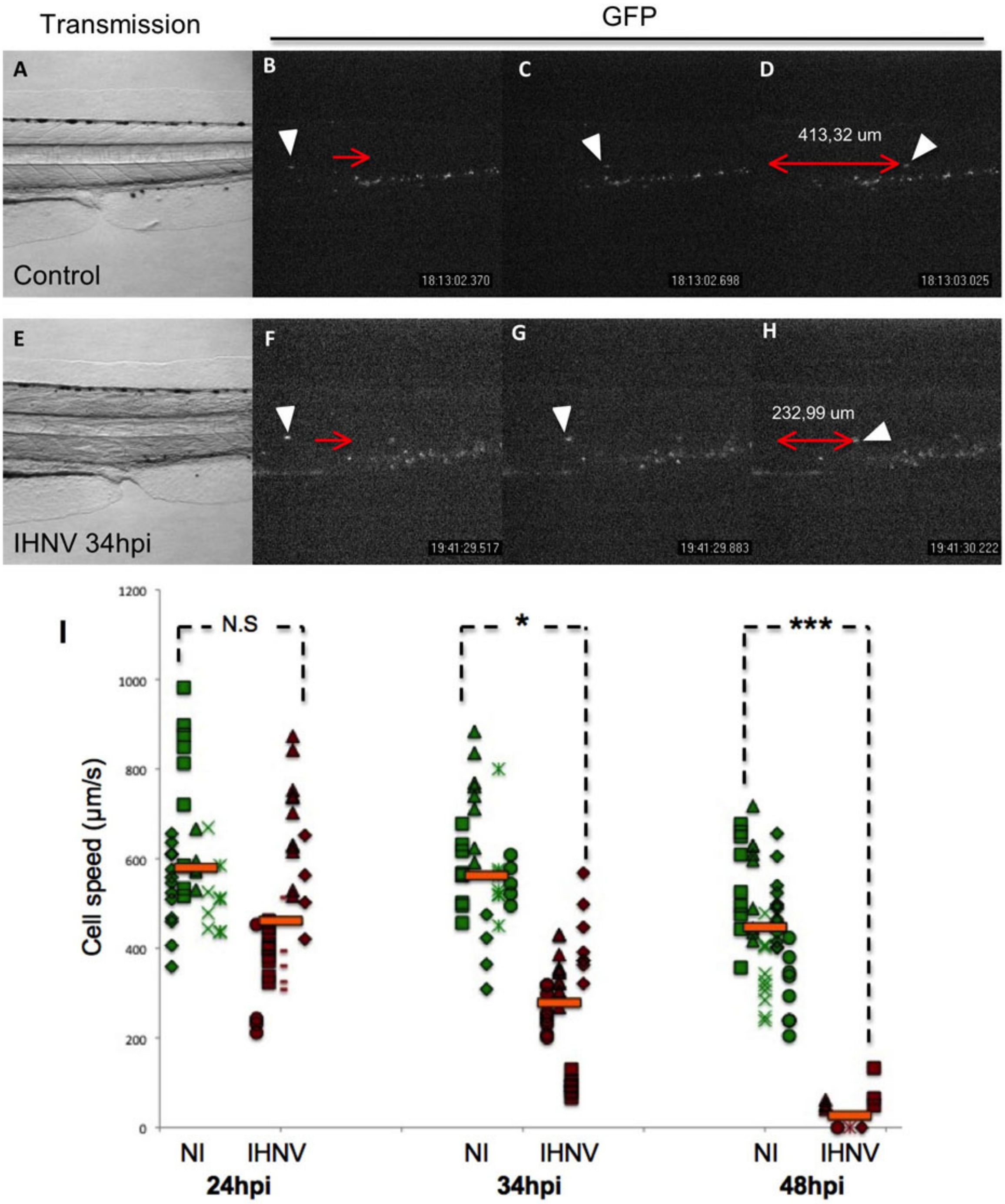

Figure S1. Progressive slowing of bloodflow during IHNV infection. A-H. Illustration of how blood speed was measured in dorsal aorta. Transmission (A,E) and green fluorescence (B-D, F-H) images of a *CD41:GFP* larva 34h after IHNV infection (hpi) (E-H) or an uninfected control (A-D), extracted from time-lapse acquisitions taken with a fluorescence stereomicroscope. Lateral views of trunk near uro-genital opening, anterior to left, dorsal to top; x6 magnification. Arrowheads show tracking of a single GFP positive thrombocyte travelling along the dorsal aorta; the immobile cells expressing GFP are hematopoietic progenitors in

the caudal hematopoietic tissue. I. Cell speeds measured in control (green marks) and IHNV-infected (brown marks) larvae at 24, 34 or 48 hpi. *n*=5 larvae in all groups except IHNV 34hpi (*n*=4). Each individual larva is represented by a specific marker shape; the speed of 5 to 15 cells has been measured in each larva, from which an individual blood speed (larva blood speed) was calculated as the average of individual cell speeds. The orange dash represents the average of larval blood speeds of each group. Statistical significance was evaluated with two-tailed Student's *t* test, considering unequal variances: N.S: not significant, $p\text{-value} > 0,05$; *: significant, $0,001 < p\text{-value} < 0,05$; ***: highly significant, $p\text{-value} < 0,001$.

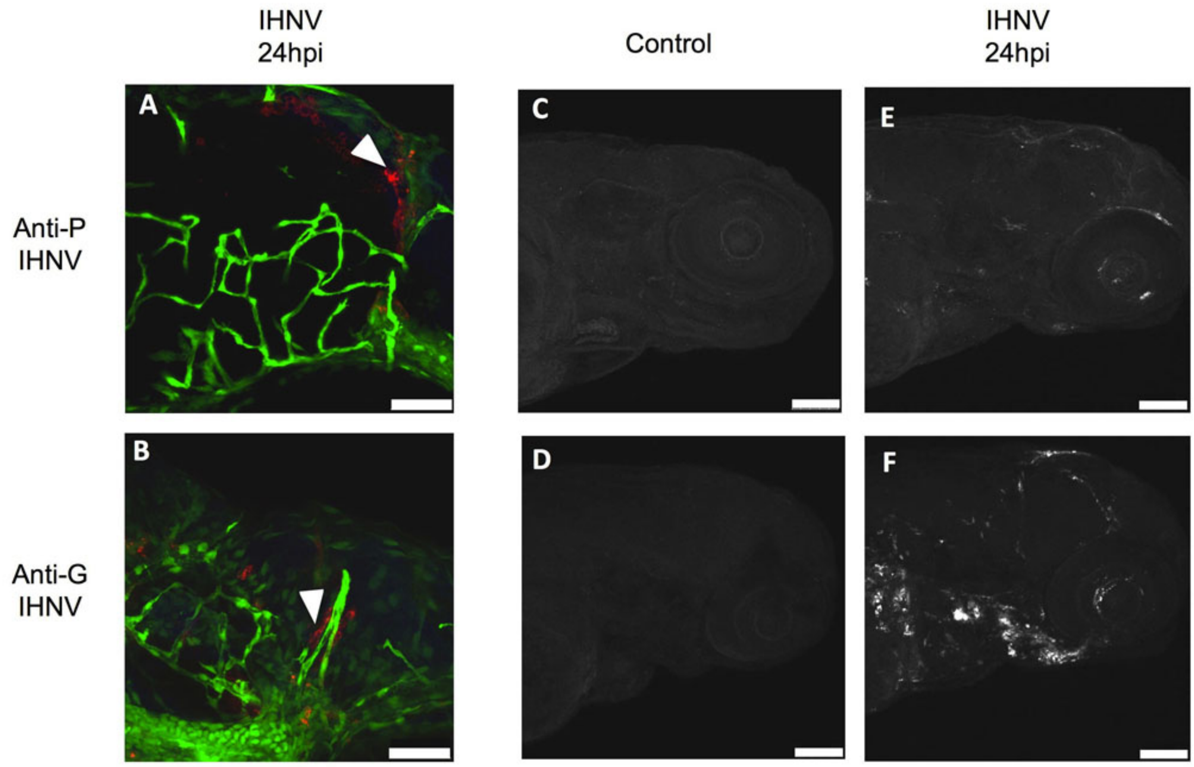

Figure S2. IHNV infection patterns are similar using anti-P or anti-G mAbs. Confocal images (maximal projections form multiple Z-stacks) of whole-mount IHC staining of *fli1:GFP* larvae. Head lateral views, anterior to right, dorsal to top. A, B, 40x objective (scale bar, 50 µm); C-F 16x objective (scale bar, 100 µm). A, B, E, F: IHNV-infected larvae fixed at 24 hpi; C, D: unifected control larvae. Colours in A and B: Red: IHNV P-protein (A) or IHNV G-protein (B); green: GFP. A and B: brain region of two larvae, showing similar pattern of IHNV infection. Notice in A and B P-IHNV protein staining and loss of GFP expression in brain vessels (white arrowheads). C-F: P-IHNV (C and E) or G-IHNV (D and F) protein staining. G-IHNV mAb is more sensitive and gives less background than P-IHNV mAb but pattern of infection are similar in both (E and F).

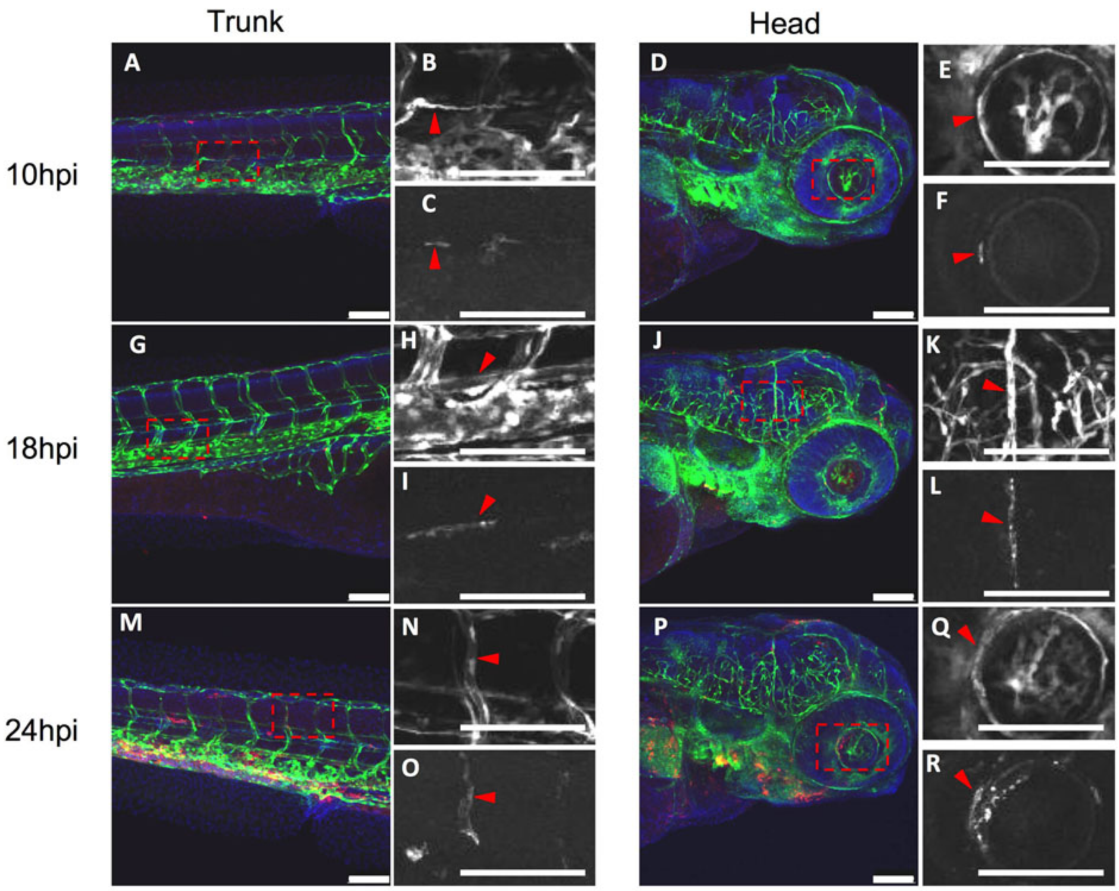

Figure S3. IHNV infects endothelial cells as early as 10hpi. A-R, confocal images (maximal projections from multiple Z-stacks) of whole-mount IHC staining of *fli1:GFP* larvae fixed 10h (A-F), 18h (G-L), or 24h (M-R) after IHNV infection; lateral views of trunk (A-C, G-I, M-O) or head (D-F, J-L, P-R), anterior to right, dorsal to top. Colours in A, D, G, J, M and P: Red: IHNV G-protein; green: GFP; blue: nuclei. To demonstrate co-localization of GFP and IHNV protein, images with overlaid signals are shown in A, D, G, J, M and P; specific GFP signal in B, E, H, K, N and Q; IHNV-G protein staining in C, F, I, L, O and R. Single GFP or G-IHNV images correspond to area inside the red box on each respective image. Red arrowheads point to cells showing colocalization of GFP and G-IHNV. C and I: IHNV infection is seen in GFP-positive cells (B and H) belonging to the dorsal aorta. O: notice IHNV infection in inter-somitic vessel with attenuated GFP expression (N). F and R: IHNV infection in the vessel circling the lens (E and Q). L: IHNV infection in the posterior cerebral vein. 16x objective; scale bar, 100 µm.

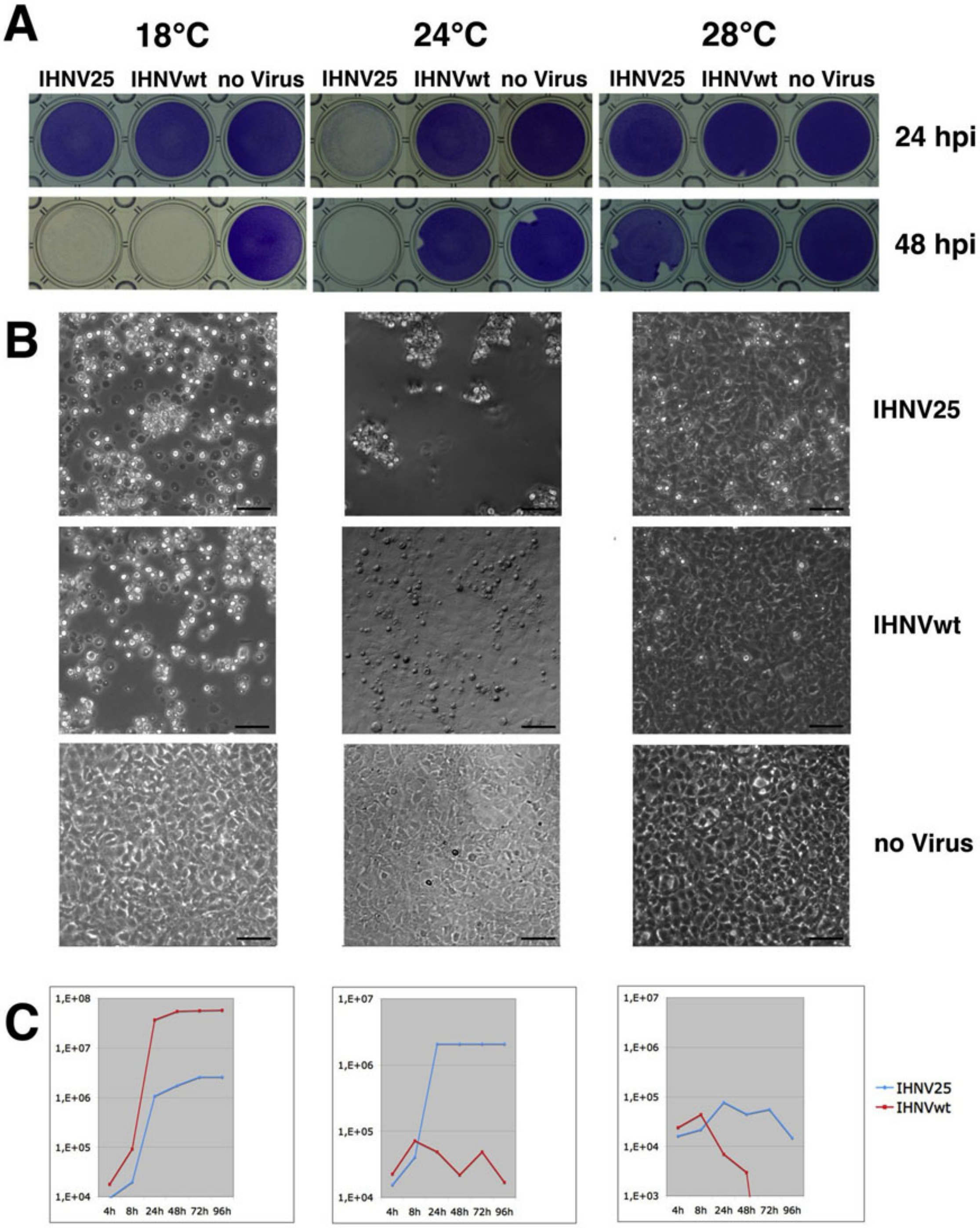

Figure S4. Temperature-dependent growth and cytopathic effect of IHNV25 and IHNVwt. A, confluent EPC cells were infected by IHNV25 or IHNVwt (MOI=1) or let without virus as a control, and grown at 18°C, 24°C or 28°C. At 24 or 48 hpi cell monolayers were fixed and stained with crystal violet. B, representative fields of live cells in duplicate wells were pictured by phase contrast microscopy to show the cytopathic effect induced by the virus. The bar represents 50µm. C, culture supernatants from cells infected with IHNV25, IHNVwt or non infected were collected at 4, 8, 24, 48, 72 and 96 hpi and the viral titer was

determined by plaque assay on EPC cells. Titers of IHNV25 are about 1000 times higher than for the wild type virus at 24°C. The titers reached by IHNV25 in these experimental conditions (MOI=1, 24°C) were never very high, which was due to a quick destruction of the cell monolayer preventing efficient virus production, as shown in panel A.
